# Distribution characteristics of intestinal flora in patients with OSAHS and the relationship between different intestinal flora and sleep disorders, hypoxemia and obesity

**DOI:** 10.1007/s11325-024-02992-8

**Published:** 2024-01-15

**Authors:** Guofei Feng, Pan Zhuge, Yaping Zou, Zhifeng Zhang, Jiandong Guo, Junxiang Ma

**Affiliations:** Department of ENT, Jinhua Central Hospital, No. 365, East Renmin Road, Jinhua City, 321000 Zhejiang China

**Keywords:** Intestinal flora, Intestinal microecology, Obstructive sleep apnoea hypopnea syndrome, Abundance

## Abstract

**Objective:**

To investigate the distribution characteristics of intestinal flora in patients with obstructive sleep apnoea hypopnea syndrome (OSAHS) of different severities and the relationship between different intestinal flora and sleep structure disorder, hypoxemia and obesity.

**Methods:**

A total of 25 healthy volunteers and 80 patients with OSAHS were enrolled in this study. The control group was healthy, and the experimental group comprised patients with OSAHS. The apnoea–hypopnea index (AHI), minimum saturation of peripheral oxygen (SpO_2min_), mean saturation of peripheral oxygen, body mass index, maximum apnoea time and other indicators were collected in clinical practice. The patients with OSAHS were divided into 20 mild and 42 moderate OSAHS cases, as well as 18 patients with severe OSAHS according to the AHI classification. Bioinformatics-related statistics were analysed using the QIIME2 software, and clinical data were analysed with the SPSS 22.0 software.

**Results:**

The changes in microbial alpha diversity in the intestinal flora of patients with OSAHS showed that richness, diversity and evenness decreased, but the beta diversity did not change significantly. The *Thermus Anoxybacillus*, *Anaerofustis*, *Blautia*, *Sediminibacterium*, *Ralstonia*, *Pelomonas*, Ochrobactrum, *Thermus Sediminibacterium*, *Ralstonia*, *Coccidia*, *Cyanobacteria*, *Anoxic bacilli* and *Anaerobes* were negatively correlated with AHI (*r* = −0.38, −0.36, −0.35, −0.33, −0.31, −0.29, −0.22, −0.18) and positively correlated with SpO_2_min (*r* =0.38, 0.2, 0.25, 0.22, 0.24, 0.11, 0.23, 0.15).

**Conclusion:**

Some bacteria showed a significant correlation with clinical sleep monitoring data, which provides a possibility for the assessment of disease risk, but the mechanisms of their actions in the intestinal tract are not clear at present. Further research and observations are needed.

## Introduction

Obstructive sleep apnoea-hypopnea syndrome (OSAHS) is a type of sleep-disordered breathing disease that seriously affects the quality of sleep and life of patients and is typically accompanied by sleep apnoea and daytime sleepiness syndrome. There are many causes of OSASHS, including obesity, excessive drinking, irregular eating habits and laryngeal diseases [1–3]. Recent studies demonstrated that OSAHS can impair the function of target organs in various systems of the body and is significantly associated with systemic diseases such as cerebrovascular disease, metabolic syndrome and tumours [4, 5]. The literature reports bacteraemia and toxaemia caused by intestinal flora translocation as primary reasons for the malignant development of stress reactions, and sleep deprivation can affect the intestinal flora of rats and promote *Clostridium perfringens* proliferation of harmful gases [6]. A growing body of research has shown gut microbiota disorders to be associated with non-communicable diseases such as cerebrovascular disease, obesity and inflammatory bowel disease; accordingly, the relationship between intestinal microecology and human health and disease is becoming a prominent direction in clinical research [7, 8]. The development of modern lifestyles and changes in dietary habits has seen OSAHS, with its high incidence rate, low awareness and numerous complications, place enormous mental burdens and economic pressure on people as a result of its health impacts [9, 10].

At present, chronic intermittent hypoxia (CIH), as a characteristic physiological change occurring in patients with OSAHS, significantly affects the structure and abundance of intestinal flora [11]. Okubo et al. [12] established a mouse model of OSAHS by CIH. Their results showed that the diversity of intestinal flora had changed; the structure of intestinal flora had also changed and indicated a decrease in Bacteroidetes and Proteobacteria and an increase in Firmicutes. Ma Jing et al. [13] of Peking University first reported the characteristics of intestinal flora in patients with OSAHS. The results showed that the number and structure of intestinal flora were increased in patients with OSAHS overall; *Clostridium* increased in patients with OSAHS, while the reduction of *Clostridium* in patients with hypertension suggests that this decrease may be one of the mechanisms of OSAHS leading to hypertension. However, the characteristics of intestinal flora in patients with OSAHS of different severities have not been further reported, and there are few reports on this direction at home and abroad.

The intestinal flora of patients with OSAHS, healthy adults and patients with moderate or severe OSAHS after surgical treatment were studied to further clarify and compare the intestinal flora composition of patients with OSAHS and healthy adults. Finally, this study aimed to explore the relationship between intestinal flora and sleep structure, hypoxemia and obesity in patients with OSAHS and to further study which intestinal flora may cause changes in the condition of patients with OSAHS to provide a theoretical basis for new intervention methods in clinical treatment in the future.

## Materials and methods

### Research subjects screening

#### Subjects

A total of 25 healthy volunteers (*N*) and 80 patients with OSAHS were enrolled in this study; the 80 patients were selected from patients with OSAHS who had been diagnosed in our hospital between January 2021 and January 2022. According to the apnoea–hypopnea index (AHI) classification, patients with OSAHS were divided into mild (L, *n* = 20), moderate (M, *n* = 22), severe (S, *n* = 18) and severe with complications (Sc, *n* = 20) groups. The population characteristics (age, sex, body mass index [BMI], diet type) of each group were matched, stool samples were collected and high-throughput sequencing was performed; bioinformatics and statistical methods were used to analyse the sequencing data of the participants’ intestinal flora. Conducting this study was given clinical research approval by the ethics committee of our hospital.

#### Inclusion criteria

The inclusion criteria for patients with OSAHS were as follows: (1) patients with light, moderate and severe OSAHS with an AHI score ≥5 times/h were diagnosed by polysomnography (PSG) throughout the night. PSG is an important method for the diagnosis of OSAHS and is conducted to determine whether patients are complicated with OSAHS by monitoring sleep structure, sleep efficiency, number of awakenings, respiration and other objective indicators; (2) the patients voluntarily signed the informed agreement and were willing to undergo follow-up; (3) the patients had no history of major surgery or severe infection in 1 year; (4) no malignant tumour and hypothyroidism; (5) no history of taking antibiotics and probiotics in the past 3 months; (6) no history of gastrointestinal disease.

#### Exclusion criteria

The exclusion criteria were as follows: (1) patients younger than 18 years or older than 80 years; (2) serious complications in every system; (3) a history of probiotic and antibiotic treatment within 3 months; (3) those who did not meet the follow-up conditions; (4) incomplete clinical data; (5) the presence of other diseases that affected the metabolism of blood glucose and blood lipids; (6) other sleep disorders or diseases that caused poor sleep patterns/quality.

#### Data collection of participants

The participant data were divided into general and clinical data. The clinical data included the following: systolic blood pressure (SBP), diastolic blood pressure (DBP), AHI, minimum saturation of peripheral oxygen (SpO_2min_), mean saturation of peripheral oxygen (SpO_2mean_) and maximum apnoea time (T_max_).

### 16s rDNA high-throughput sequencing

#### Stool specimen collection

A sterile faeces collector was used to collect fresh faeces samples, which were labelled properly and stored in a −80 °C freezer.

#### Extraction of total microbial DNA from stool specimens

Using the OMEGA Soil DNA Kit (D5625-01), total genomic bacterial DNA was extracted from the samples and stored at −20°C. The quantification and assessment of the extracted total DNA’s quantity and quality were performed using the NanoDrop ND-1000 spectrophotometer (Thermo Fisher Scientific, Waltham, MA, USA) and 1.2% agarose gel electrophoresis.

#### The 16S rRNA gene was amplified by polymerase chain reaction

The highly variable V3-V4 region of the bacterial 16S rRNA gene, approximately 468 base pairs long, was selected for sequencing. Polymerase chain reaction (PCR) amplification of the V3-V4 region of the bacterial 16S rRNA gene was conducted using forward primer 338F (5′-barcode+ACTCCTACGGGAGGCAGCA-3′) and reverse primer 806R (5′-GGACTACHVGGGTWTCTAAT-3′). The PCR started with an initial denaturation at 98 °C for 2 min. Then, the amplification cycles were conducted: 98 °C denaturation for 15 s, 50 °C annealing for 30 s and 72 °C extension for 30 s, repeated 25 times. Finally, a 5-min extension was performed at 72 °C, followed by storage at 4°C. The target fragment was isolated using 2% agarose gel electrophoresis and recovered with the Axygen Gel Recovery Kit (BioTek, Beijing, China).

#### Purification, quantification and mixing of PCR amplification products

The PCR amplification products were purified using Agencourt AMPure Beads (Beckman Coulter, Indianapolis, IN). The quantification of PCR products was performed using the Quant-iT PicoGreen dsDNA Assay Kit in a microplate reader (BioTek, FLx800), and then the samples were mixed according to the required data volume for each sample.

#### TruSeq library construction

The sequencing library was prepared using Illumina’s TruSeq Nano DNA LT Library Prep Kit. The specific steps were as follows:

1. The amplified products underwent sequence end repair. The End Repair Mix 2 in the kit was used to remove protruding bases at the 5′ end of the DNA sequence while adding a phosphate group and filling in any missing bases at the 3′ end. Additionally, an A base was added to the 3′ end of the DNA sequence to prevent the self-connection of DNA fragments and to ensure compatibility with the sequencing adapter (which has a protruding T-base at its 3′ end).
2. Sequencing adapters containing library-specific labels (i.e. index sequences) were added to the 5′ end of the sequences, enabling DNA molecules to be immobilised on the flow cell.
3. Beckman AMPure XP Beads were used to purify the library system after the addition of adapters, eliminating self-ligated adapter fragments through magnetic bead selection.
4. The DNA fragments attached to the adapters were amplified by PCR to enrich the sequencing library templates. The library enrichment products were purified once again using Beckman AMPure XP Beads.
5. The final step involved fragment selection and purification of the library using 2% agarose gel electrophoresis.

#### Illumina NovaSeq sequencing

1. Prior to sequencing, the library underwent quality checking on the Agilent Bioanalyzer using the Agilent High Sensitivity DNA Kit. A qualified library displayed a single peak and lacked adapter dimers.
2. The library was quantified using the Quant-iT PicoGreen dsDNA Assay Kit on the Promega QuantiFluor® fluorescence quantification system. The concentration of a qualified library should be above 2 nM.
3. Qualified sequencing libraries (with non-repetitive index sequences) were gradually diluted and mixed in the appropriate proportions based on the required sequencing volume. They were denatured using NaOH to become single-stranded before being subjected to sequencing.
4. Double-ended sequencing was performed using the NovaSeq sequencer and employing the NovaSeq 6000 SP Reagent Kit (500 cycles).

#### Bioinformatics analysis

1. Original sequencing data: Paired sequencing of microbial DNA fragments was conducted using the Illumina NovaSeq platform. The DADA2 method was utilised to optimise sequence data processing (noise removal or clustering) on the Qiime2-LRB-2019.4 bioinformatics analysis platform.
2. Utilising the QIIME2 (2019.4) bioinformatics analysis platform involved primarily employing two methods: DADA2 and VSEARCH. The denoising approach, with DADA2 as the representative method for generating feature sequences, is currently recommended by major analysis platforms (e.g. QIIME2 and USEARCH). DADA2 (Benjamin et al., 2016) involves processes such as primer removal, quality filtering, denoising, joining and chimera removal. It does not perform similarity clustering but focuses on dereplication, which is equivalent to clustering at 100% similarity. Each unique sequence generated after DADA2 quality control is referred to as ASVs (amplicon sequence variants) or feature sequences (equivalent to OTU representative sequences). The abundance of these sequences across samples forms a feature table (similar to an OTU table). The ASV feature sequences and tables from each library were analysed separately and merged, removing singleton ASVs (i.e. sequences occurring only once in the entire dataset by default) to obtain high-quality sequences. Finally, the sequencing depth for each sample was calculated. As DADA2 is currently not compatible with all amplicon types, the VSEARCH method, based on OTU clustering (Rognes et al., 2016), is retained as an alternative. The VSEARCH method involves processes like primer removal, joining, quality filtering, dereplication, chimera removal and clustering sequences at a default 97% similarity level, forming different representative OTU sequences and OTU tables. It eliminates singleton OTUs (i.e. OTUs with an abundance of 1 in all samples by default) and their representative sequences and assesses the sequencing depth for each sample.

#### Standardisation of ASV/OTU

To standardise the sequencing depth across all samples, the ASV/OTU tables require rarefaction processing. This can be accomplished using a sparse (rarefaction) method by randomly extracting a specific number of sequences from each sample to reach a uniform depth, predicting the ASVs or OTUs observed in each sample at said sequencing depth and their relative abundance (Heck et al., 1975; Kemp and Aller, 2004). In QIIME2 (2019.4), the “qiime feature-table rarefy” function is used, setting the rarefaction depth to 95% of the lowest sample sequence count. Subsequently, the rarefied ASV/OTU statistical tables were used for various analyses such as diversity, abundance and the structural composition of gut microbiota.

### Statistical analysis

The bioinformatics data were analysed by Qiime2 software, and the clinical data were analysed using SPSS 22.0 software. The Shapiro–Wilk test was used to test the normality of the quantitative data. The data obeying normal distribution are statistically described by mean ± standard deviation, and the *t*-test is used for between-group comparisons. Data that did not obey the normal distribution are described by median or interquartile range, and a non-parametric Kruskal–Wallis H test was used for between-group comparison. The count data is statistically described by the number of cases or a percentage, and the between-group comparison was compared using a chi-square test; *P* < 0.05 was considered statistically significant.

## Results

### Basic information of the participants

There was no significant difference in age, sex and diet type among the mild, moderate and severe OSAHS group and the healthy group (*P* > 0.05). There were significant differences in BMI, SBP and SBP among the healthy group and the mild, moderate and severe OSAHS groups (*P* < 0.05). There were significant differences in AHI, T_max_, SpO2_min_ and SpO2_mean_ among the mild, moderate and severe OSAHS groups (*P* < 0.05) (see Table 1).

**Table 1:** Basic characteristics of the subjects studied

Index	N (*n* = 25)	L (*n* = 20)	M (*n* = 22)	S (*n* = 18)	Sc (*n* = 20)	*P*
Age (years)	40.31±2.56	45.38±3.62	49.45±2.98	44.35±3.12	48.27±2.37	>0.05
Male (%)	20 (80)	17 (85)	36 (85.7)	14 (77.8)	27 (90.0)	>0.05
Diet type						>0.05
Carnism	8	7	7	5	6	
Vegetarian	9	7	9	6	5	
Balanced diet	8	6	6	7	9	
BMI (kg/m^2^)	22.53±0.35	23.65±0.52	26.73±0.65	29.36±0.73	29.02±0.53	<0.05
SBP (mmHg)	118.46±2.20	124.32±3.52	125.33±3.25	136.56±2.35	136.60±2.54	<0.05
DBP (mmHg)	75 (70, 85)	77 (75, 90)	83 (76, 91)	89 (85, 96)	89 (85, 98)	<0.05
AHI (Times/hours)		6.9 (6.1, 10.5)	18.7 (17.9, 20.1)	60.3 (58.6, 61.3)	80.13 (74.20, 113.37)	<0.05
T_max_ (s)		16.50 (12, 26)	26.3 (19, 31.5)	67.3 (58, 79)	82.75 (59.2, 97.71)	<0.05
SpO_2min_ (%)		85 (81, 89)	80 (75, 84)	74 (69, 80)	71 (61, 78)	<0.05
SpO_2mean_ (%)		94 (93, 95)	93 (92, 94)	91 (89, 93)	90 (87, 94)	<0.05

*N*, health; *L*, mild; *M*, moderate; *S*, severe; *Sc*, severe with complications; *BMI*, body mass index; *SBP*, systolic blood pressure; *DBP*, diastolic blood pressure; *AHI*, apnoea-hypopnea index; *SpO*_*2min*_, minimum saturation of peripheral oxygen; *SpO*_*2mean*_, mean saturation of peripheral oxygen; *T*_*max*_, maximum apnoea time

### Alpha diversity analysis

**Fig. 1:**
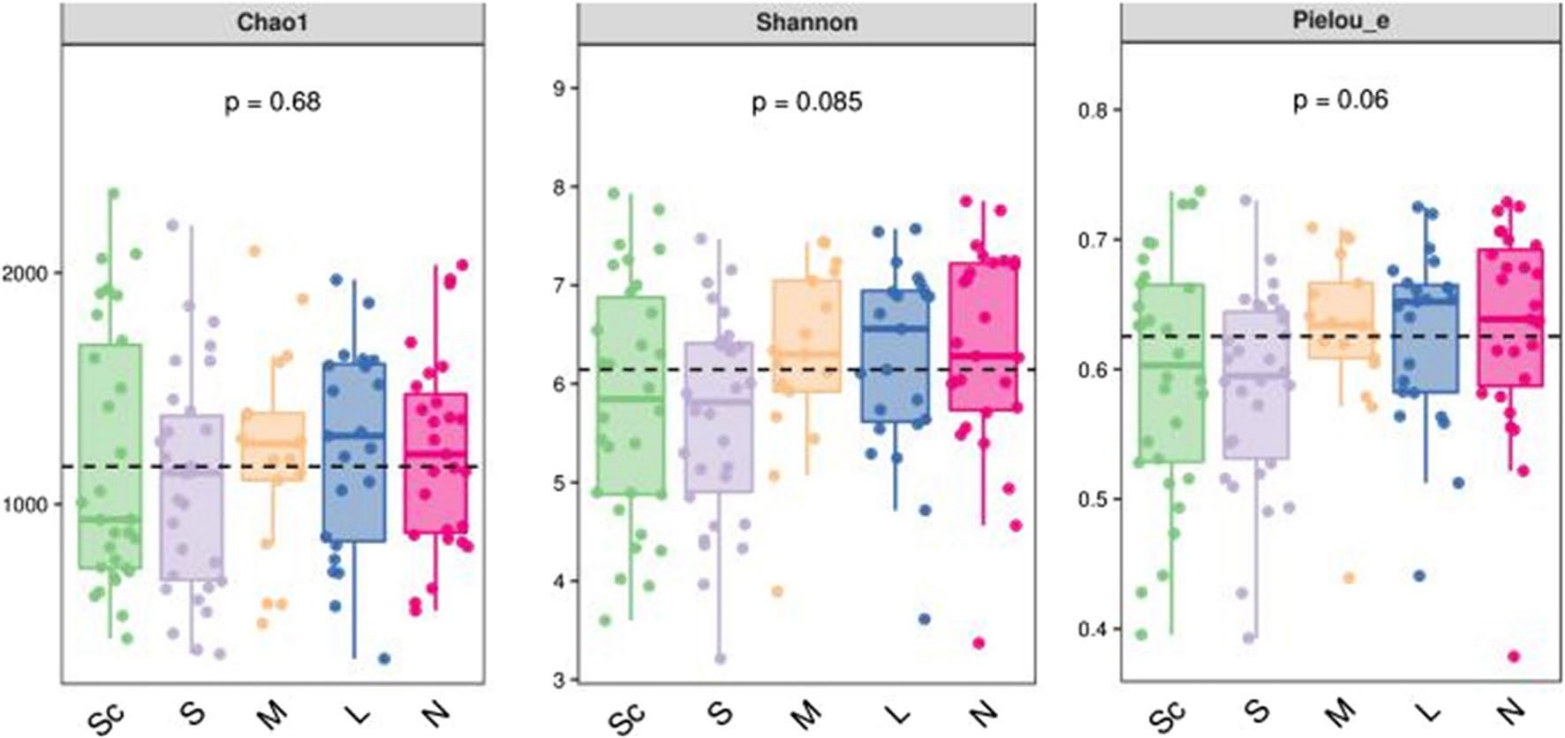
Alpha diversity analysis boxplot. Note: N, health; L, mild; M, moderate OSAHS; S, severe OSAHS; Sc: severe with complications OSAHS

Alpha diversity is an indicator of the richness, diversity and evenness of the microbial community in a sample. We used the Chao1 index to represent richness, the Shannon index to represent diversity and Pielou’s evenness index to represent evenness. Based on the ASV table, QIIME2 software was used for analysis, and the results showed that with the aggravation of OSAHS in patients, the Chao1, Shannon and Pielou’s evenness indexes decreased to some extent, but there was no significant difference (*P* > 0.05) (see Fig. 1).

### Beta diversity analysis

**Fig. 2:**
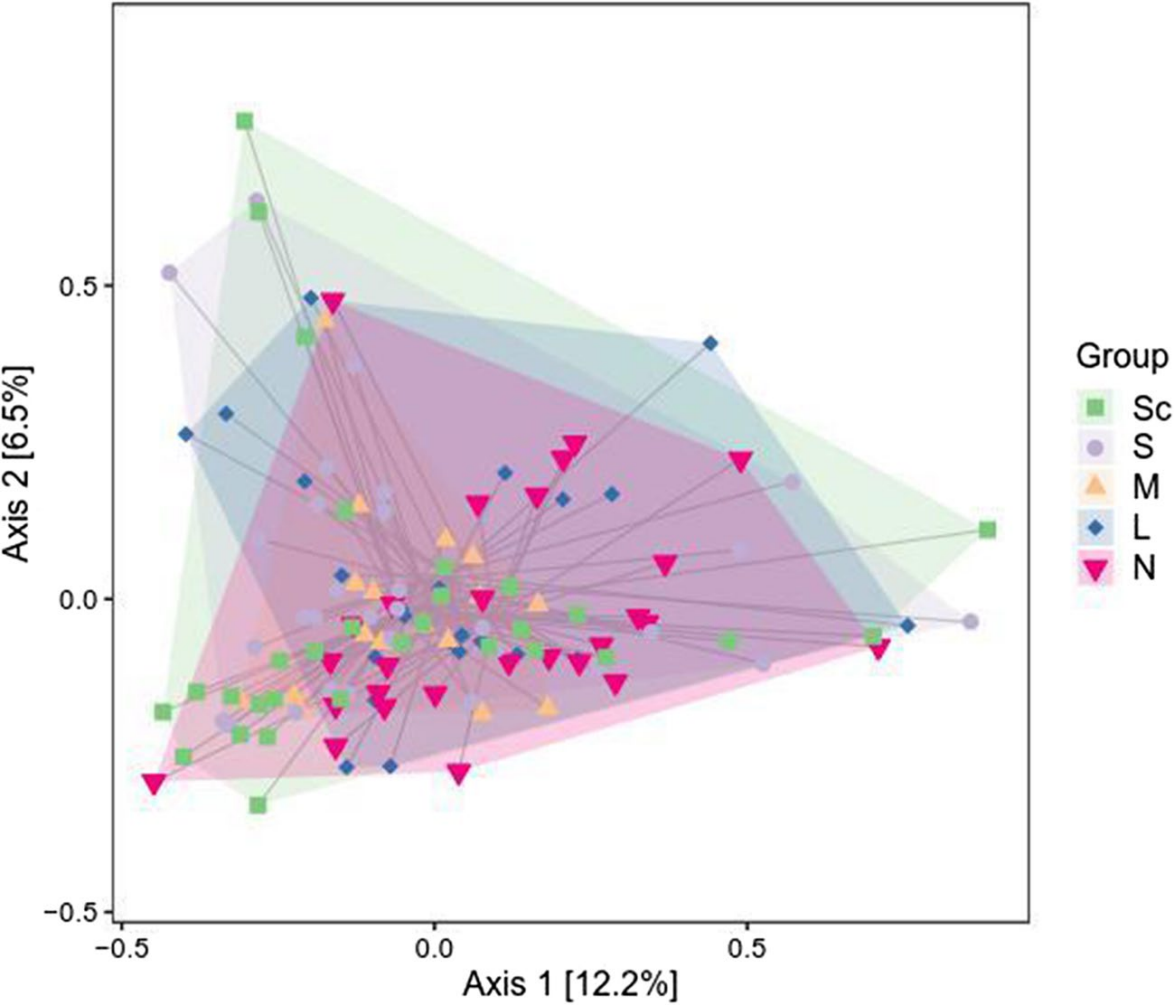
Beta diversity analysis profile. Note: N, health; L, mild OSAHS; M, moderate OSAHS; S, severe OSAHS; Sc, severe with complications OSAHS. The significant difference in distance between groups was analysed by Adonis difference analysis. *P* = 0.22

The abundance information of ASV species in the flattened samples and the evolutionary relationship between sequences were used. Concurrently, the weighted UniFrac distance algorithm in the QIIME2 software was used to reduce the measurement of multi-dimensional species data to create the sample difference distance, and the distance matrix was constructed. We further understand the differences in the overall level of intestinal flora structure and composition in patients with OSAHS with different severity. A large number of distance matrices were sorted into two dimensions using the principal coordinate analysis (PCoA) method, and the final results were visualised. The PCoA analysis employed the distance formed by the vertical projection of species in the corresponding coordinates as a method to measure the difference in composition. The smaller the projection distance, the more similar the species composition was (Fig. 2).

### Statistics of taxonomic units

**Fig. 3:**
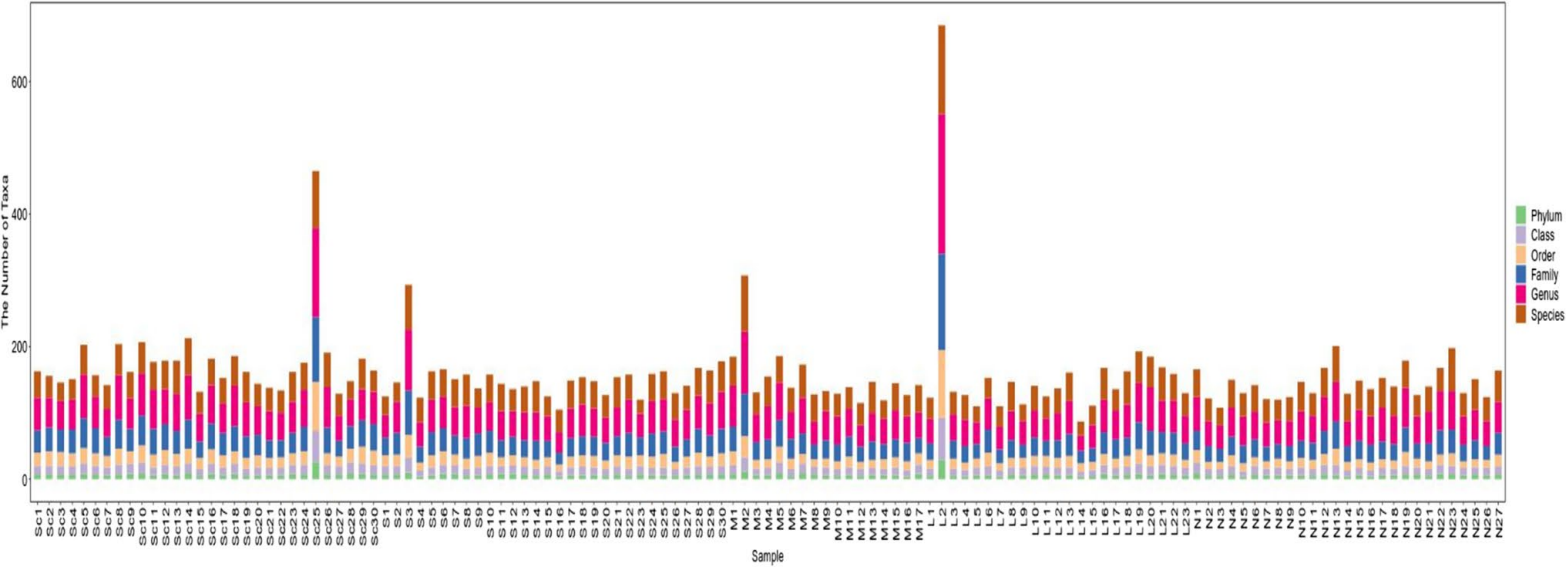
Histogram of taxonomic unit statistics. Note: The horizontal coordinates are all sample names; the vertical coordinates are phylum, class, order, family, genus and species; six levels respectively contain the number of taxa

Based on the flattened ASV statistical table, the distribution of microbial communities in each sample at each taxonomic level was obtained as shown in Fig. 3.

### Correlation analysis of intestinal flora and sleep monitoring indexes

To further explore the association of gut microbiota with polysomnography, we statistically analysed BMI, AHI, SpO_2min_, SpO_2mean_ and T_max_ in patients with OSAHS; the results of Spearman correlation analysis between intestinal flora and these indicators are shown in Table 2. Based on the results, the abundance of *Shigella* (*R* = −0.17) and *Brucella* (r = −0.19) were negatively correlated with BMI, while the abundance of *Actinomyces* (*R* = 0.32) and *Akkermansia* (*r* = 0.21) were positively correlated with BMI. *Thermus*, *Sediminibacterium*, *Ralstonia*, *Pelomonas*, *Blutia, Anoxybacillus* and *Anaerofustis* were negatively correlated with AHI (*r* = −0.38, −0.31, −0.29, −0.22, −0.33, −0.36 and −0.35, respectively) and positively correlated with SpO_2min_ (*r* = 0.23, 0.11, 0.24, 0.23, 0.22, 0.20 and 0.25), and the difference was statistically significant (*P* < 0.05) (see Table 2).

**Table 2:** RDA correlation analysis table of sleep monitoring indexes and intestinal flora abundance (only the part showing statistical significance)

	BMI	AHI	SpO_2min_	SpO_2mean_	*T*_max_
*Acinetobacter*	−0.12	0.32*	0.17	0.22	0.34
*Actinomycetospora*	0.32*	−0.19	−0.41	−0.10	−0.20
*Akkermansia*	0.21*	−0.24	0.22	0.19	−0.30
*Anaerofustis*	−0.39	−0.35*	0.25*	0.26	−0.40*
*Anoxybacillus*	0.35	−0.36*	0.20*	0.26	−0.40
*Bifidobacterium*	−0.21	0.38	0.23*	0.12	0.17
*Blautia*	−0.26	−0.33*	0.22*	0.23	−0.26
Brevibacillus	−0.23	−0.25	0.33	0.14	0.18
*Brucella*	−0.19*	−0.24	0.26	−0.12	−0.27
*Bulleidia*	0.21*	0.18	−0.17	0.28	0.21
*Butyrivibrio*	0.33	0.28	0.26	0.29*	0.28
*Chelatococcus*	0.15	0.37*	0.28	−0.18	0.34
*Cloacibacillus*	−0.18	0.22	0.24	0.37	0.33*
*Collinsella*	0.27	0.27*	−0.28	−0.24	0.36
*Coprobacillus*	0.33	0.12*	0.22*	−0.13	0.42
*Corynebacterium*	0.31	0.30	0.33*	−0.21	0.22
*Cupriavidus*	−0.29*	0.38*	0.24	−0.24	0.36
*Dyella*	0.19*	0.18	−0.16	0.22	0.27
*Eggerthella*	0.34*	0.31	0.25*	−0.24	0.14
*Eubacterium*	−0.28*	0.10	−0.21	−0.12	0.09
*Fusobacterium*	0.28	0.24*	−0.02	0.18	0.09
*Gemmiger*	0.28	0.28*	−0.03	0.18	0.18
*Megasphaera*	−0.18	0.22	0.31*	0.27*	0.17
*Mogibacterium*	0.31*	0.14	0.17	0.14	0.23
*Neisseria*	−0.10	0.23*	−0.14	−0.31*	−0.18
*Ochrobactrum*	0.22*	−0.18*	0.15*	0.18	0.24
*Paraprevotella*	0.24	−0.17	0.19	0.17	0.28*
*Pelomonas*	0.21	−0.22*	0.23*	−0.31	−0.23
*Peptostreptococcus*	−0.08	0.27*	0.18	−0.15	0.34
*Pigmentiphaga*	0.19*	0.18	−0.17	0.15	0.14
*Ralstonia*	−0.18	−0.29*	0.24*	−0.18	−0.31
*Rothia*	−0.29*	0.22*	0.22	0.17*	0.23
*Rubellimicrobium*	0.15	0.34*	0.24	−0.18	0.24*
*Sarcina*	0.35	0.42	0.27	−0.11*	−0.15
*Sediminibacterium*	0.11	−0.31*	0.11*	−0.23	−0.24
*Shigella*	−0.17*	−0.25	−0.40	0.02	−0.27
*Succinispira*	0.24	0.36*	0.14	0.08	−0.07
*Sulfitobacter*	0.21*	0.17	0.17	0.18	0.24
*Thermus*	0.39	−0.38*	0.23*	0.35	−0.22
*Turicibacter*	−0.18*	−0.17	0.25	0.22	0.04

*P < 0.05; *BMI*, body mass index; *AHI*, apnoea-hypopnea index; *SpO*_*2min*_, minimum saturation of peripheral oxygen; *SpO*_*2mean*_, mean saturation of peripheral oxygen; *T*_*max*_, maximum apnoea time

## Discussion

In recent years, the relationship between intestinal microecology and human health and disease has become an important issue in social and clinical research [14]. To date, many studies at home and abroad have confirmed the close relationship between intestinal microflora disturbance and disease and have explored its influence on disease from the perspective of intestinal microecology, targeting disease-specific interventions as a new therapeutic direction [15, 16]. However, the bulk of studies on the relationship between OSAHS and intestinal microflora have focused on animals because of the large number of intestinal microflora. The specific CIH pathological state of OSAHS is closely related to intestinal microflora; however, few studies have been conducted on the relationship between OSAHS and intestinal microflora [17]. On the one hand, many factors affect the intestinal flora, including congenital (e.g. heredity, mode of delivery) and acquired (e.g. diet, living habits, surgery, antibiotics) factors. On the other hand, it is difficult to study the relationship between intestinal flora and patients with OSAHS in clinical research, which requires long periods of time to follow-up. Furthermore, in current research, in the context of whether the intestinal flora is related to the occurrence and development of OSAHS, and if it participates in patients with OSAHS with multi-system injury, the particular mechanism that may be involved is unclear.

In this study, we used high-throughput sequencing technology to analyse the characteristics of patients with OSAHS of different severities, based on what is reflected in a large number of studies. The results showed no significant difference in the diversity and abundance of intestinal flora between patients with OSAHS and healthy cohorts, and there was no significant difference in alpha diversity between patients with OSAHS and healthy individuals. At the same time, the beta diversity of intestinal flora was also analysed to observe changes in intestinal flora composition and structure in patients with OSAHS of different severities. Based on the results, there was no significant difference between patients with OSAHS and the healthy group or between patients with OSAHS and the self-group. However, this conclusion is not consistent with previous animal studies [18]. Accordingly, a large number of clinical studies are needed to verify these results.

Based on the above findings, the present study carried out species composition relationships at the phyla and genus levels for the intestinal flora of patients with different OSAHS severity levels. The results showed that the intestinal flora that were present represented the phyla level. The dominant flora comprised mainly Firmicutes, Bacteroidetes, Proteobacteria and Fusobacteria, among which Firmicutes and Bacteroidetes accounted for more than 90%. In healthy people, the ratio of Firmicutes to Bacteroidetes is often considered a sign of a healthy gut, whereas an increase in the Firmicutes/Bacteroides (F/B) ratio caused by an increase in Firmicutes or a decrease in Bacteroidetes is considered to be a marker of obesity-and-hypertension-related intestinal dysregulation [19, 20]. Leyre et al. [21], in contrast to the analysis of gut microbes in healthy people, showed that the abundance of Firmicutes significantly increased in obese individuals, and Bacteroidetes decreased by approximately 90% in comparison with healthy individuals; with dietary interventions, the F/B ratio gradually returned to the normal level observed in healthy individuals, and weight also decreased significantly. At the same time, it has been shown that Bacteroidetes can significantly inhibit the growth of adipocytes. The results showed that BMI and Firmicutes increased with OSAHS severity, Bacteroidetes decreased and the F/B ratio increased in patients with OSAHS. It is suggested some changes in the composition of intestinal microflora occur between patients with OSAHS and healthy individuals, that is, dysbiosis of intestinal microflora occurs in patients with OSAHS, which can be reduced by intervening with the F/B ratio or controlling the body weight of patients with OSAHS, which has become one of the ways to reduce the risk of disease [22]. Studies have reported that weight loss through lifestyle changes, anti-obesity drugs and bariatric surgery can improve OSAHS symptoms [23]. Pihtili et al. found that there were significant differences in OSAHS-related indexes between obese OSAHS patients and normal OSAHS patients, such as apnoea–hypopnea index, oxygen desaturation index, mean SpO(2) and lowest SpO2, indicating a close correlation between obesity and OSAHS [24]. Therefore, the exact relationship between obesity and OSAHS requires further exploration. The analysis of the species composition of the top 20 dominant bacterial genera and their relative abundance in intestinal flora indicated a decreasing trend for Faecalibacterium with the aggravation of OSAHS.

Polysomnography is considered the gold standard in the diagnosis of OSAHS. To explore whether some species of intestinal flora were related to the indexes of sleep monitoring, we carried out correlation analysis of BMI, AHI, SpO_2min_, SpO_2mean_ and T_max_. According to RDA analysis, there was no significant difference between AHI, SpO_2min_, SpO_2mean_ and T_max_ and the overall abundance of intestinal flora, except that BMI was positively correlated with the overall abundance of intestinal flora. Spearman correlation analysis of bacterial abundance with sleep monitoring indicators was subsequently performed and the results were as follows: (1) there was a negative correlation between Shigella abundance and brute abundance and BMI; (2) the abundance of *Actinomycetospora* and *Akkermansia* was positively correlated with BMI; (3) the abundance of *Thermus*, *Sediminibacterium*, *Ralstonia*, *Pelomonas*, *Blautia*, *Anoxybacillus* and *Anaerofustis* were negatively correlated with AHI and T_max_ and positively correlated with SpO_2min_, respectively. Respiratory *Pelomonas* is considered to be a key bacterium contributing to the pathogenesis of allergic asthma, but its mechanism of action in the gut is currently unclear [25]. *Anoxybacillus*, *Anaerofustis* and *Blautia* belong to Firmicutes, and the indexes of clinical sleep monitoring with their presence in the gut. An increase in the proportion of respiratory bacteria and anaerobic bacteria may lead to chronic immune–inflammatory disorder, which indirectly affects sleep quality by affecting the respiratory and digestive symptoms of patients. A continuous inflammatory state is also related to the occurrence and development of OSAHS. However, previous studies have not reported the impact of a pathogen on the occurrence and severity of OSAHS. Further research is needed to clarify whether this correlation is involved in the development of OSAHS and whether it has good clinical significance in predicting its severity.

There is a bidirectional relationship between sleep architecture and gut microbiota composition, and interference with gut microbiota by antibiotics leads to greater fragmentation of non-rapid eye movement (NREM) sleep, which, in turn, reduces sleep quality. Sleep disruption can also lead to changes in the gut microbiota; however, there appears to be a lack of agreement on the relationship between sleep architecture and gut flora. In the microbial–brain–gut axis system, the intestinal flora and changes in sleep structure are closely related. When the circulation in this system is restricted, macrophages can be activated based on the proportion of intestinal flora (probiotics/prebiotics) to enhance non-specific and specific immune responses and natural killer cell activity, thereby enhancing the expression level of cytokines to promote the expression of immunoglobulin, particularly secretory IgA, to improve sleep quality and structure. These findings provide an important scientific basis for conducting intestinal flora intervention in the treatment of related neuropsychiatric disorders. However, further studies are needed to clarify whether the above-noted correlations are involved in the occurrence and development of OSAHS and whether they are of guiding significance in clinical treatment.

## Conclusion

The composition and structure of intestinal flora in patients with OSAHS compared with a healthy group. The relative abundance of faecal bacteria in patients with OSAHS showed a downward trend, indicating that disorder in intestinal flora was related to the occurrence and development of OSAHS. Some bacteria showed a significant correlation with clinical sleep monitoring data, which provides the possibility for the assessment of disease risk. However, their mechanism of action in the intestinal tract is not yet clear, and further research and observations are needed in this regard.

## Data Availability

All data generated or analysed during this study are included in this article. Further enquiries can be directed to the corresponding author.
